# CNS Intravascular Lymphoma: A Case Report

**DOI:** 10.1155/2011/515182

**Published:** 2011-09-07

**Authors:** Amer Awad, Bachir Estephan, Olaf Stüve

**Affiliations:** ^1^Baton Rouge Neurology Associates, Baton Rouge General Medical Center, 3600 Florida Boulevard, Baton Rouge, LA 70806, USA; ^2^Department of Neurology, University of Kansas Medical Center, Kansas, KS, USA; ^3^Department of Neurology, University of Texas Southwestern Medical Center, Dallas, TX, USA; ^4^Department of Immunology, University of Texas Southwestern Medical Center, Dallas, TX, USA; ^5^Neurology Section, VA North Texas Health Care System, Medical Service, Dallas, TX, USA

## Abstract

Intravascular lymphoma is a rare but well-described entity. The clinical manifestations are heterogeneous. We report a case of a 59-year-old woman who presented initially with syncope followed by subacute cognitive decline that progressed to minimally conscious state. Shortly after the transfer to our tertiary center the patient died. Brain autopsy disclosed the diagnosis of B-cell intravascular lymphoma. We speculate that syncope could be the first manifestation of central nervous system intravascular lymphoma and should be considered in the differential diagnosis of unexplained syncope. In addition, we stress the importance of early brain biopsy in unexplained white matter disease.

## 1. Case Report

The patient was a fifty-nine-year-old right-handed school counselor Caucasian woman with a past medical history remarkable for left-sided breast cancer, in remission, status postresection ten years prior to admission, hypothyroidism on thyroxin, and hyperlipidemia on rosuvastatin. She was transferred to our neurology tertiary center, from a local hospital, for evaluation and treatment of unexplained altered mental status. The patient's history dates back to four months when she suffered an episode of loss of consciousness consistent with syncope. She suffered prodrome lightheadedness. Shortly after that, everything turned black and then blacked out for few minutes. During the episodes she was able to hear people talking to her but could not respond. No jerking was witnessed. In addition, there was no tongue biting or urinary incontinence. She woke up clear-headed with complete recollection of the event. The event last for about 60 seconds. At that time, she underwent cardiac evaluation which was unremarkable. 

 About two months after the syncopal episode, the patient's family and coworkers started noticing intermittent problems with her cognitive function. Specifically, she displayed difficulties using her computer. However, she continued to work but had progressive difficulties with common daily tasks. One month later, the patient was admitted to a local hospital with an acute and transient episode of dysarthria and facial palsy. A brain magnetic resonance imaging (MRI) was completed to exclude a cerebrovascular etiology of her acute symptoms. The MRI was reported as abnormal due to the presence of “white matter disease”. While in the hospital, she became progressively worse and her mental status deteriorated quickly over the course of one week. Repeat brain MRI was reported as “significantly worse” than the initial one and showed multiple areas of white matter lesions some of which showed restricted diffusion. Cerebrospinal fluid analysis was pursued to exclude intracranial infections and the only abnormality reported was a high protein level. The exact number was not available. Full anticoagulation with unfractionated heparin and coumadin was started as well as empirical treatment with acyclovir. Acyclovir was discontinued after a viral infection was excluded by cerebrospinal fluid (CSF) polymerase chain reaction (PCR) studies. The patient continued to deteriorate over the next week to a minimally responsive state. No details were available from the records we reviewed on her pre-deterioration status. A computerized tomography (CT) of the head disclosed progression of the white matter disease with interval development of a left parietal parenchymal hemorrhage, in addition to diffuse cerebral edema. Anticoagulation was reversed, and she was transferred to our hospital for higher level of care.

Upon admission to our institution the patient was in a minimally conscious state, which allowed only a limited neurological examination. She was arousable to voice and followed one-step commands inconsistently. She was able to repeat and answer only simple questions inconsistently as well. Pupils were equal and reactive. Corneal reflexes were present bilaterally. Visual fields were intact to threat. No gross restriction of extraocular muscle movement was noticed. No gaze deviation of skew deviation was noted. No nystagmus was observed. Her face was symmetric. The tongue was midline with no atrophy or fasciulations. Motor examination was consistent with a mild-moderate spastic pyramidal left hemiparesis. No involuntary movements were seen. Muscle stretch reflexes were brisk (3^+^) bilaterally. Plantar reflexes were extensor bilaterally. The patient was able to localize tactile and noxious stimuli on both sides without obvious side-to-side difference.

**Figure 1:**
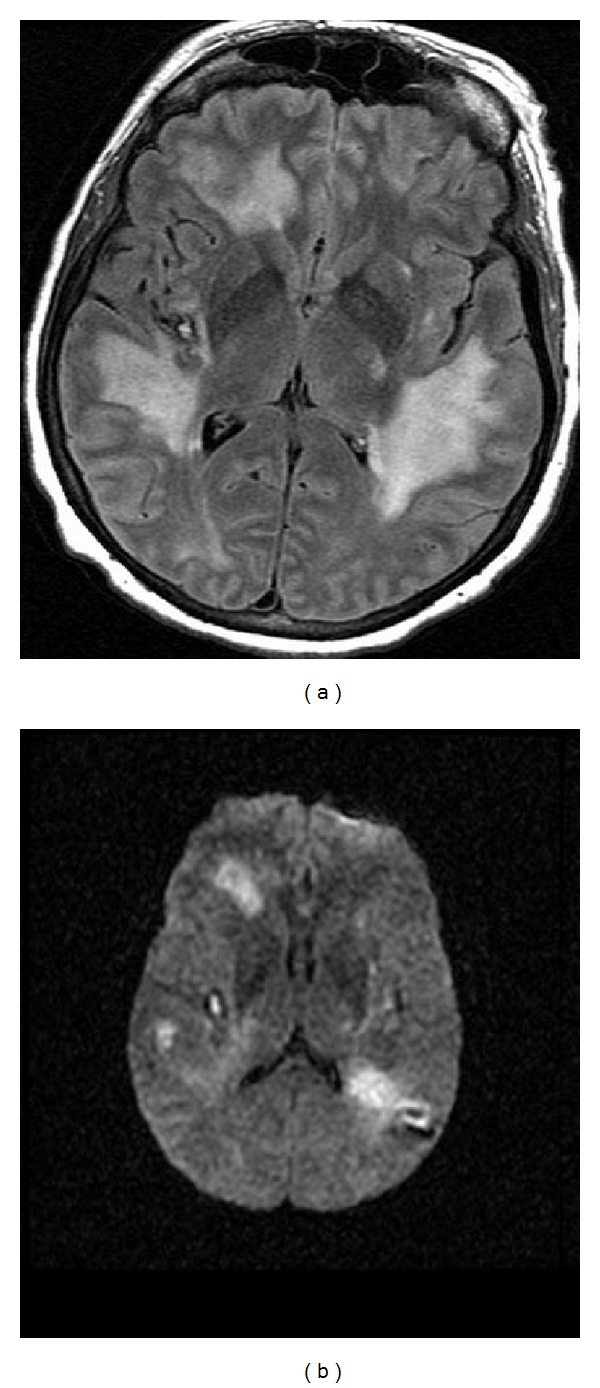
Multiple confluent white matter lesions demonstrated by 3 mm axial fluid attenuated inversion recovery (FLAIR) brain magnetic resonance images (MRIs) demonstrating and diffusion weighted imaging (DWI), respectively.

A complete blood count was remarkable for anemia and thrombocytopenia (hemoglobin 9.5 mg/dL and platelet count of 83,000/mm^3^respectively). Results of a basic metabolic panel, which included serum electrolytes, blood glucose, and kidney-function tests, were within normal limits. Liver function tests revealed elevated liver enzymes: alanine aminotransferase 82 IU/L, aspartate aminotransferase 96 IU/L; albumin low 2.8 mg/L; ammonia elevated to 57 mg/L. Inflammatory markers were abnormal: erythrocyte sedimentation rate 36 and C-reactive protein 1.9. Antinuclear antibody was positive with a low titer of 1 : 320. Other pertinent normal/negative studies: coombs, platelet antibodies, heparin-induced thrombocytopenia antibodies, lupus anticoagulant antibodies, antiphospholipid antibodies, hepatitis C and B, copper, vitamin B12, homocysteine, D-dimer, CA-125, CA 19-9, West Nile virus IgG/IgM, serum protein electrophoresis, thyroid stimulating hormone, extractable nuclear antigens, and antineutrophil cytoplasmic antibody. CSF examination showed a protein of 90 with 4 white blood cells and glucose 59. A herpes simplex virus polymerase chain reaction was negative. Brain MRI showed multiple white matter confluent lesions correspondent to restricted diffusion on diffusion-weighted imaging (DWI) (Figure 1). A magnetic resonance angiography (MRA) of the head and neck was normal. 

Empiric treatment with high dose (1 gm daily) intravenous methylprednisolone for 5 days and anticonvulsants was initiated. Lactulose therapy was started, as well, as the patient was found to have transaminitis and hyperammonemia.

Her neurological status did not improve despite the normalization of hepatic parameters. A brain biopsy was considered, but she became clinically unstable due to interval development of several medical complications, including transaminitis, thrombocytopenia, renal insufficiency, and upper extremity deep venous thrombosis. A needle biopsy of the left breast mass showed only a fibrotic tissue and excluded a malignant process. The patient deteriorated rapidly, developed pyuria and pneumonitis and died, three weeks after her transfer to our hospital, of fulminant respiratory failure. The patient's family agreed to pursue brain autopsy to investigate the etiology of her neurological symptoms and rapid clinical deterioration. 

**Figure 2:**
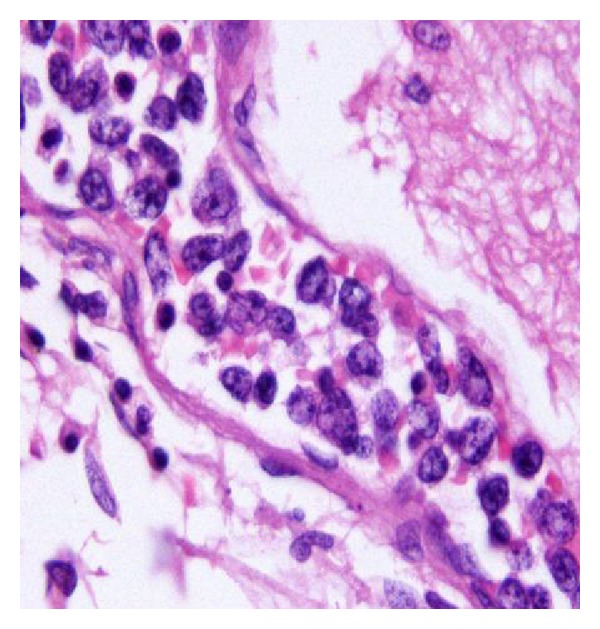
Brain biopsy demonstrating the presence of intravascular atypical lymphocytes.

Gross examination of the brain showed multiple hemorrhagic lesions throughout the cerebrum. Histopathological examination shown in Figure 2 confirmed the diagnosis of B-cell intravascular lymphoma (angiotropic lymphoma). The atypical lymphocytes were found to be CD20/CD79a^+^.

## 2. Discussion

Intravascular Lymphoma (IVL) (Angiotropic Lymphoma) is a rare and heterogeneous subtype of lymphoma. The neoplasm was initially thought to be of endothelial origin and used to be known as “angioendotheliomatosis proliferans systemisata,” “angioendotheliomatosis,” or “intravascular endothelioma” [1–3]. However, it was not until the early 1980's when a lymphatic origin was established and confirmed by multiple clinicopathological studies [4–7]. The overwhelming majority of IVL are of B-cell origin [8, 9]. Intravascular lymphoma, as the name implies, is typically confined to the intravascular spaces, however, extravascular involvement has been rarely reported [10, 11]. This unique subtype of lymphoma does spare the primary lymphatic tissue in striking comparison to systemic lymphomas [11, 12]. From a clinical standpoint, IVL is considered a “great masquerader” as it usually presents with a myriad nonspecific symptoms involving multiple systems, more commonly nonspecific neurological symptoms, namely, mental status changes and dementia, nonspecific skin rashes, unexplained fever, and adrenal masses [9, 13]. A very high index of suspicion is important to diagnose this rare entity pre-mortally. Unfortunately, the diagnosis is not uncommonly missed prior to brain autopsy. 

Intravascular lymphoma, though rare, is a well-described clinical entity with heterogeneous clinical manifestations. However our case has a unique “sentinel” presentation which is syncope. The clinical presentation is inconsistent with seizure. Theoretically, we do not expect the patient with subcortical white matter lesions to present with seizures. 

To our knowledge no previous reports had documented syncope as the earliest manifestation of IVL. The premortal diagnosis of IVL remains a major challenge to clinicians and our case report should help alert physicians and neurologists to include IVL in the differential diagnosis of unexplained syncope. The pathogenesis of syncope secondary to intravascular lymphoma is probably vascular. From a cerebrovascular standpoint, syncope can either result from vertebrobasilar insufficiency or bilateral internal carotid artery insufficiency. Theoretically, IVL can cause an embolic transient ischemic attack (TIA). The embolus in the case consists of tumor cells rather than blood clot. 

This case also underlines the importance of early brain biopsy in patients with unexplained atypical white matter disease. Paucity of risk factors of vascular disease, younger age, and the MRI atypical confluent distribution of white matter disease should alert neurologists to purse brain sampling early in the course of the disease. 

We also propose that brain MRI should be considered in cases with unexplained syncope. The usual workup utilized brain CT which is inadequate tool to evaluate nonhemorrhagic brain lesions.

## 3. Conclusion

Intravascular lymphoma is a rare and fatal disease that presents with a myriad of nonspecific symptoms. To ascertain the earliest diagnosis possible, and to potentially alter patients' outcomes, we propose to include intravascular lymphoma it as a differential diagnosis in patients presenting with syncope of unclear etiology. Brain MRI should be considered in evaluating patients with unexplained syncope. Finally, the cases raises the importance of early brain biopsy in unexplained white matter disease which could positively impact the course of a progressive fatal disease.
